# Cross-infection and infection control in dental clinics in Nablus and Tulkarm districts

**DOI:** 10.1186/s12866-021-02382-0

**Published:** 2021-12-20

**Authors:** Wafaa Menawi, Areej Sabbah, Lubna Kharraz

**Affiliations:** 1 Public Health Management Program, Faculty of Graduate Studies, An-Najah University, Nablus, Palestine; 2 Pathology and Medical Laboratory Sciences Department, Medicine and Health Science college, An Najah National University, Nablus, Palestine; 3 Head of Quality Assurance Department at NBU, Scientific Centers, An Najah National University, Nablus, Palestine; 4 Master of Public Health Management, Faculty of Graduate Studies, An-Najah University, Nablus, Palestine; 5 Medicine and Health Science College, An Najah National University, Nablus, Palestine

**Keywords:** Infection control, COVID 19, PPE, Compliance, Knowledge, Palestine, Dentists

## Abstract

**Background:**

Infection control had many developments in the COVID 19 (Coronavirus Disease 2019) pandemic, despite this, there were many complications in different health care facilities as well as dentists’ clinics due to the lack of infection control knowledge and compliance failure. This study aimed to assess the level of knowledge and compliance with the infection control measures in the dental clinics in the Nablus and Tulkarm districts.

**Results:**

The results showed that the total positive response regard all infection control domains were (70.0 %). Whereas the participants gave the highest positive response for personnel protective equipment i.e. gloving was (96.10 %). They gave the instruments related to controls the lowest responses, i.e. instruments sterilization was (59.40 %). The analyzed data showed significant statistical differences in the compliance with infection control measures between Nablus and Tulkarm districts “p < 0.05” in the interest of dentists from Tulkarm.

**Conclusions:**

In conclusion, the findings of this study showed that there is moderate compliance to infection control protocol in Nablus and Tulkarm dental clinics. Thus, there is a need to strengthen adherence to infection control measures.

**Method:**

A universal sampling was used to assess the infection control program at the dental clinics in Nablus and Tulkarm Districts. The study sample involved 265 dentists. Data was collected using a questionnaire which has been sent via email between July and August 2020. Descriptive statistics, Chi-square test, One-way ANOVA and Post-Hock tests have been used. Statistical significance was set at ″P <0.05″. Cronbach’s alpha has been conducted to ensure the reliability and validity of the questionnaire.

## Background

Dental care practices are not risk-free [1]. A white-coat, a dental instrument, and a dental unit are susceptible to the splatter of blood, aerosol and saliva, trauma, or inoculation by contaminated instruments [2]. Thus, cross-infection and infection in dental clinics have become a major public concern [3]. As an action, health care practitioner needs to understand how are infectious diseases transmitted to minimize the risk [4]. Subsequently, appropriate safety precautions should be taken within the dental environment to prevent cross-infection transmission among patient-patient or patient-dental staff [5]. Today, the globe lives the ghost of Corona Virus disease, and it is the most appropriate time to emphasize the importance of cross-infectious disease and infection control measures [6]. In developing countries, infection control measures are either not documented, not followed, not funded by the government, or not existed, which makes the challenge of acquiring an infection during health care delivery increase in those countries [7]. Many studies have revealed that the neglecting of safety measures can cause adverse events and lead to hospital admission [8], increase in medical expenditure, permanent injuries, or even death [9]. These adverse events also can expose the dentist to a legal accountability allegation in courts, scandals of the press, and loss of money to compensate the affected patient if malpractice and substandard health care are applied in addition to the loss of license of the dental practice, while these adverse events could be manageable and avoidable [9]. Several factors may affect the compliance of dental practitioners to infection control measures. It could be the degree of education or knowledge [10], lack of incentives and costs [11], professional variables, socio-demographic, availability of infection control equipment, or access to this equipment [12].

The first manifestation of some infectious diseases appears as lesions in the oral cavity as TB (Tuberculosis), HIV (Human Immunodeficiency Virus), Syphilis, and Hepatitis [13] and COVID 19 [6]. The dental practitioner should have enough knowledge about these diseases to take precautions during dental care procedures [14]. The dental staff should implement Source Control Measures (SCM). Those measures, include hand hygiene, respiratory hygiene, cough etiquette, safe injection practices, safe handling of potentially contaminated equipment, and personnel protective equipment [15], in addition to maintaining a six-foot distance between patients in the waiting room to prevent the spread of the SARS-CoV-2 virus (Severe Acute Respiratory Syndrome Coronavirus) [16]. In Palestine, the obstacles impede the enjoyment of the highest attainable standards of health for Palestinians living under occupation, including barriers and lack of access to adequate health care [17]. A study conducted in the north of Palestine showed that visiting dental clinics and doing dental procedures are considered as the most significant risk factors for the acquisition of HBV (Hepatitis B Virus) infection [18]. But actually, the real estimation of cross-infection and infection control measures in these clinics is not clear as well as these measures are not controlled by the Palestinian Ministry of Health (PMH).

In this study, the authors evaluated the level of knowledge and compliance with standard precautions of infection control in dental clinics related to Nablus and Tulkarm districts, Palestine.

## Results

### Participants characteristics

As shown in Table 1, nearly two-thirds (58.9 %) of participants were males. The majority were non-specialists (75.1 %), and (56.2 %) were from Nablus district. A fifth of the participants (21.5 %) have been in dental practice for less than 5 years as well as being those practicing between 11 and 15 years and (14.3 %) of total participants have been practicing for more than 20 years. Finally, eighty-nine (89.1 %) of the total respondents were working in private clinics, (9.1 %) in governmental clinics and the least (1.8 %) were working in the united nations relief  and works agency (UNRWA).

**Table 1:** Participants Characteristics

Gender	No.	%
M	156	58.9
F	109	41.1
Total	265	100.0
Educational level	No.	%
Specialist	66	24.9
General	199	75.1
Total	265	100.0
Governorate	No.	%
Nablus	149	56.2
Tulkarm	166	43.8
Total	265	100.0
Years of experience	No.	%
≤5	57	21.5
6-10	64	24.2
11-15	60	22.6
16-20	46	17.4
>20	38	14.3
Total	265	100.0
Ownership	No.	%
Private	236	89.1
Public	24	9.1
UNORWA	5	1.8
**Total**	**265**	**100.0**

### Infection control protocol assessment domains

Table 2 showed that the total compliances’ regard all infection control domains mentioned in the study were (70.0 %). Whereas the participants gave the highest positive response for personnel protective equipment, gloving (96.10 %), face masking (77.70 %), protective clothing (76.30 %), hand washing (76.10 %), vaccination against HBV (74.50 %) and eye protection (74.30 %). They gave the instruments related to controls the lowest responses; instruments sterilization were (59.40 %), and aerosol control, accident management, and monitoring autoclave were (55.1 %, 55.30 %, and 47.20 %) respectively except the surface decontamination with the responses of (78.00 %).

**Table 2:** Positive Response Percentage for Intended Domains

NO.	Intended Domain	Positive Response %
1.	Hepatitis vaccination	74.50 %
2.	Wearing Gloves	96.10 %
3.	Wearing a face mask during dental procedures	77.70 %
4.	Wearing eye protection	74.30 %
5.	Wearing Protective Clothing, head cap, and white coat	76.30 %
6.	Hand washing	76.10 %
7.	Instruments Sterilization	59.40 %
8.	Monitoring Autoclave	47.20 %
9.	Decontamination and Cleaning surfaces, using disposable protection parries to cover some surfaces	78.00 %
10.	Aerosol Control	55.1 %
11.	Accident Management	55.30 %
**Average of Positive Response Percentage for Intended Domains.**		**70.0 %**

### Compliance of participants with infection control protocol according to governorates

Data analysis by T-test clarified that there were significant differences between the two Governorates (Nablus and Tulkarm) ″p < 0.05″ in seven domains; wearing gloves, wearing protective clothing, a head cap, and a white coat, hand washing, instruments sterilization, decontamination, and cleaning surfaces, using disposable protective barriers to cover some surfaces, aerosol control, and accident management. All these significant differences were in favor of Tulkarm versus Nablus governorate by referring to the means for the seven domains mentioned in Table 3 below.

**Table 3:** Compliance of Participants with Infection Control Protocol According to Governorate

Domain	Governorate	N	Mean	S.D	T	P-value
Hepatitis vaccination	Nablus	149	0.72	0.304	1.381	0.168
	Tulkarm	116	0.77	0.190		
Wearing gloves	Nablus	149	2.74	0.437	3.282	*0.001
	Tulkarm	116	2.89	0.204		
Wearing a face mask during dental procedures	Nablus	149	2.12	0.805	1.179	0.239
	Tulkarm	116	2.23	0.715		
Wearing eye protection	Nablus	149	1.99	0.771	1.272	0.204
	Tulkarm	116	2.10	0.703		
Wearing Protective Clothing, head cap, and white coat	Nablus	149	2.11	0.635	2.121	*0.035
	Tulkarm	116	2.26	0.525		
Hand washing	Nablus	149	2.19	0.575	3.851	*0.000
	Tulkarm	116	2.44	0.422		
Instrument sterilization	Nablus	149	0.57	0.203	3.293	*0.001
	Tulkarm	116	0.65	0.169		
Monitoring autoclave	Nablus	149	0.45	0.348	1.278	0.202
	Tulkarm	116	0.50	0.353		
Decontamination and cleaning surfaces, using disposable protection barriers to cover some surfaces	Nablus	149	2.15	0.647	3.269	*0.001
	Tulkarm	116	2.39	0.552		
Aerosol control	Nablus	149	0.51	0.302	2.694	*0.008
	Tulkarm	116	0.61	0.287		
Accident management	Nablus	149	0.65	0.336	2.967	*0.003
	Tulkarm	116	0.76	0.251		

T-Test ,*p-value<0.05

### Compliance of participants with infection control protocol according to ownership

The ANOVA F test (Table 4) showed that there were significant differences in wearing a face mask during a dental procedure, eye protection, monitoring autoclave and aerosol control domains attributed to the ownership variable ″P < 0.05″, the Post-Hoc test showed that the UNRWA group with means of 1.40,1.00, 0.13, 0.27 respectively were lower than all other groups

The same table presented that there were significant differences in wearing protective clothing, hand washing and decontamination and cleaning surfaces, using disposable protection barriers to cover some surfaces domain attributed to the ownership variable ″P < 0.05″ and the Post-Hoc test showed that the private group with means of 2.21, 2.36 and 2.32 orderly were higher than all other groups

**Table 4:** Compliance of Participants with Infection Control Protocol According to Ownership

Domain	Ownership	N	Mean	S.D	F	p-value
Hepatitis vaccination	Private	236	0.74	0.265	0.394	0.675
	Public	24	0.78	0.248		
	UNRWA	5	0.80	0.112		
Wearing Gloves	Private	236	2.81	0.365	0.692	0.501
	Public	24	2.76	0.350		
	UNRWA	5	2.65	0.224		
Wearing a face mask during dental procedures	Private	236	2.17	0.757	0.3443	*0.033
	Public	24	2.38	0.770		
	UNRWA	5	1.40	0.894		
Wearing eye protection	Private	236	2.08	0.722	5.993	*0.003
	Public	24	1.88	0.850		
	UNRWA	5	1.00	0.000		
Wearing Protective Clothing, head cap, and white coat	Private	236	2.21	0.584	7.599	*0.001
	Public	24	2.01	0.577		
	UNRWA	5	1.27	0.149		
Hand washing	Private	236	2.36	0.502	15.046	*0.000
	Public	24	1.90	0.500		
	UNRWA	5	1.55	0.447		
Instruments Sterilization	Private	236	0.61	0.194	1.606	0.203
	Public	24	0.59	0.183		
	UNRWA	5	0.46	0.039		
Monitoring Autoclave	Private	236	0.49	0.350	4.758	*0.009
	Public	24	0.33	0.326		
	UNRWA	5	0.13	0.183		
Decontamination and cleaning surfaces, using disposable protection barriers to cover some surfaces	Private	236	2.32	0.573	16.928	*0.000
	Public	24	1.92	0.729		
	UNRWA	5	1.00	0.000		
Aerosol Control	Private	236	0.56	0.300	3.087	*0.047
	Public	24	0.49	0.278		
	UNRWA	5	0.27	0.149		
Accident Management	Private	236	0.70	0.311	1.203	0.302
	Public	24	0.67	0.282		
	UNRWA	5	0.50	0.000		

(ANOVA) F test, *p-value<0.05

## Discussion

### HBV vaccination

Moderate compliance (74.50 %) was recorded toward HBV vaccination between participants in both districts. A similar study was conducted in Jordan (2020) and found that (82.1 %) of dental health care providers were compliant with vaccination against hepatitis B [19]. The current study revealed that there were no significant differences based on socioeconomic characteristics regarding HBV vaccination. Particularly, we are living under the greatness of the COVID 19 outbreak, so the medical teams wherever need to be armed with strong immunity as well as possible.

### *PPE* (Personal Protective Equipment)

In our study, the compliance with personal protective equipment was between high and moderate i.e. gloving was (96.10 %). In the same field, compliance with gloving among dentists was lower in Hebron (2017) (69.95 %) [20] and here we can say that knowledge and compliance are better among dental practitioners in Nablus and Tulkarm than those in Hebron or may be interpreted by COVID 19 epidemic as dentists in GAZA recorded high gloving compliance (98 %) [21] like Nablus and Tulkarm. The study also revealed that there were significant differences in wearing gloves attributed to the governorate variable (P-value<0.05). The T-test showed that the Nablus district has a mean of (2.74) which is lower than the Tulkarm district (2.89). This means high knowledge about the importance of gloving among Tulkarm dentists maybe because of the awareness activities which were done by the dental association- Tulkarm branch to enhance the educational situation among its dentists. Also, results exhibited less adherence to mask -like in Gaza (70 %) [21] than Lebanese dentists which was recorded (89.1 %) in 2017 [12]. Also, the current study indicated significant differences in wearing a face mask during dental procedures attributed to the ownership variable (P-value<0.05). The Post-Hoc test showed that the UNRWA group, including the mean of (1.40) was the lowest of all other groups. This result means that there is a problem in dental care provided by UNRWA dental clinics, as these clinics provide dental services for a large number of refugees in more than five camps in two governates. Here, competent committees from (PMH) or from UNRWA camps need to enforce the infection control system. We are still focusing on PPE, the compliance with eye protection is moderate among participants in the current study (74.30 %). This means fair knowledge about the importance and indicators for eye protection, compared with participants in a study that had been done among a group of military dentists in April 2009. As (50.57 %) never used eyeglasses or protective face shields [22]. A study in Hebron in 2017 revealed that only (12.8 %) of dental practitioners were compliant with eye protection [20], and (32 %) in Gaza [21].

The use of protective clothing, a head cap and a white coat is very important during dental care procedures. The study of Nablus and Tulkarm showed that (76.30 %) of the participants comply with Wearing Protective Clothing, head cap, and white coat.

Generally, the compliance with all (PPE) measures among the participants was (81.1 %) which means that there is a serious awareness of the global COVID 19 pandemic that we are all living till now. Khan and Chughtai (2020) come out that (HCWs) uses gloves and face masks more than any other (PPE) to protect them from infections and respiratory diseases. Thus, overall compliance and attitudes to the use of PPE were low [23]. The Nablus – Tulkarm study indicated significant differences in wearing eye protection that attributed to the governorate variable ″P < 0.05″. Where T-test showed the that Nablus group with a mean of (2.11) lower than the Tulkarm group with a mean of (2.26). These readings illustrate the need to encourage the competent committee in the Nablus district to provide more knowledge to dental practitioners about infection control measures. The study also revealed that there were significant differences in wearing protective clothing, a head cap, and a white coat attributed to the ownership variable ″P < 0.05″. The Post-Hoc test showed that the mean of private clinics is higher than other groups. This may underline that the dentist in the private clinic is assiduous to appear in a good appearance in front of clients.

### Hand washing

In the Nablus-Tulkarm study, the overall attended hand washing is moderate among participants (76.10 %). This is a low result compared with a study had been done in Jordan which revealed that hand washing after treatment was (83.2 %) and prior to starting treatment was (66.3 %) and about one-half (45.8 %) usually reported washing hands before wearing gloves [19]. The current study reported significant differences in handwashing attributed to the governorate variable (P-value<0.05). The T-test showed that the Nablus district has a lower meaner (2.19) than Tulkam with a mean of (2.44). Again, significant differences in handwashing attributed to the ownership variable ″P < 0.05″ were seen. The Post-Hoc test showed that UNRWA with a mean of (1.55) lower than other groups. This could be because of the high work pressure on UNRWA dental clinics and the huge numbers of patients visiting these dental clinics, causing a shortage of time to apply hand washing carefully after each dental task.

### Instrument sterilization

Autoclaving is the most effective one of instrument sterilization in the dental field [24]. This way of sterilization received a high degree of compliance (94 %) among participants in Nablus and Tulkarm districts while the total instrument sterilization (decontaminant solution, washer disinfector, antiseptic and wrapping bags) was (59.4 %). This result is high compared with another one in a study among Lebanese dentists in 2017 which showed that steam autoclaving is the preferred means of sterilization (65 %) [12]. Another study was conducted in Hebron-Palestine in 2017 highlighted that the response regarding instrument sterilization was relatively low (42.8 %) where the level of compliance according to Sterilization and Disinfection of Patient Care Tools (SDT) was very high 88 % ^20^.

The Nablus- Tulkarm study also revealed that there were significant differences in instrument sterilization attributed to the governorate level variable ″P < 0.05″. The T-test showed that the Tulkarm group with a mean=0.65 is higher than the Nablus group with a mean of (0.57). These results support what has been said previously that Tulkarm dentists have good knowledge about the means of infection control measures and they are keen to apply these measures more than Nablus dentists. This may be due to better awareness activities were done by the competent committees in Tulkarm district. These activities included medical conferences, regular and periodic inspections on dental clinics as Tulkarm district, or medical meetings, including lectures to raise the awareness of applying (ICM).

Significant differences in instrument sterilization attributed to the educational level variable ″P < 0.05″ have been documented. The T- test showed that the specialist group with a mean=0.55 was lower than (GP) group with a mean=0.62. This means good knowledge among (General Practitioner) group about the importance of sterilization and at the same time seriousity in the application of this method is more than in the specialist group. It also revealed that there were significant differences in instrument sterilization attributed to the governorate level variable ″P < 0.05″.

### Autoclave monitoring

In general, autoclave monitoring means that dentists should stop work completely in case of malfunction.

The result of the current study reflected fair knowledge about the ways of sterilization as they recorded (70.9 %) for the familiarity of autoclave monitoring methods, while they recorded a low positive response regarding the evaluation of the autoclave using chemical and biological as a whole (47.20 %). A significant difference in monitoring autoclave attributed to the ownership variable ″P < 0.05″. The Post-Hoc test of the private group with a mean (0.49) was higher than other groups. This result may emphasize that they are assiduous to appear in a good appearance in front of their patients besides the practitioner.

### Decontamination and cleaning surfaces, using disposable protection barriers

Environmental surfaces are those that do not come in contact directly with patients, but they can play a major role in transmitting the pathogens [25]. This study showed that a moderate percentage (78.0 %) of participants do decontamination and clean surfaces and use disposable protection barriers to cover some surfaces, a high percentage (91.7 %) of them used disinfectant to clean surfaces away from patient contact between patients, comparing that with other dentists in which they used disinfectant agents by (28.9 %) [26]. Our study has a high positive result in this field that mirrored a high awareness and knowledge about the importance of disinfection in the dental environment. Also, (80.0 %) of all participants regard covering surfaces that can’t be decontaminated which in another study were used by (70.2 %) of private dental clinics [19]. The result of this study also reflected good compliance to cleaning and disinfection. As (68.00 %) of all participants cover light cure with special bags and (73.30 %) of them use disposable protection barriers to cover the dental unit chair. These two environmental surfaces are highly touched by dentists and staff hands. So it can be the main source of bacterial transmission. This study showed that (77.0 %) of all participants discard the disposable protection barriers after finishing the procedure. This moderate result is better than the result documented by Idris (2012) in which none of the study dentists used plastic barriers to cover the clinical contact surfaces [27]. These moderate responses have to be increased by enhancing knowledge among dentists and their staff and by informing patients about the curiosity of seeing the dentist or the assistant changes these disposal barriers. The study analysis exhibited that there were significant differences between Nablus and Tulkarm dental clinics in favor of Tulkarm clinics in decontamination and cleaning surfaces and using disposable protection ″P < 0.05″. Tulkarm clinics (Mean=2.39) have decontamination and cleaning and using disposable protection more than Nablus clinics (Mean=2.15). These repeated results among all infection control domains confirm that Tulkarm’s dental clinics are superior over those of Nablus’s in terms of commitment to applying infection control measures, this commitment may come from high knowledge among Tulkarm dentists or continuous and frequent inspection of these dental clinics by stakeholders (dental association- Tulkarm branch or (PMH). There were also significant differences in decontamination and cleaning surfaces and using disposable protection barriers to cover some surfaces among participants attributed to the ownership variable ″P < 0.05″. The Post-Hoc test showed that UNRWA dental clinics with a mean (1.00) were the lowest group in applying decontamination compared with other dental clinics. This result enforces what we noticed in the handwashing domain that when there is a large number of clients and patients visiting clinic per day (5 working hours). This will lead to drowse in order to disinfect and clean between patients.

### Aerosol control

All dental care using dental handpieces can form aerosol and splatter which are commonly impured with bacteria, fungi, viruses or blood [28]. A rubber dam is one of the many ways that can prevent the spreading of contaminated aerosol during dental procedures [29]. The participants of this study showed low compliance regard using rubber dams (31.30 %), but they were better than dentists in other studies which indicated (23.8 %) [30] and (2.4 %) [27] usage of rubber dams. The low percentage in the current study can be because of low knowledge about the importance of such a device. So, a highly concentrated effort should be made by competent committees’ regard to using a rubber dam. The High-Volume Evacuator is another method was used during dental care procedures to prevent aerosol contamination by suctioning a large amount of blood and saliva that is secreted during headpiece working [28]. The participants of the current study showed a high degree of compliance with (HVE) (86.80 %), this is a good result compared with another one in which (28.6 %) of public hospitals use high volume evacuation hospitals compared to (19.4 %) in academic institutions [19]. Another study revealed that (61.6 %) did not use high vacuum suction [27]. The good result may come from the point that (HVE) is connected with a dental chair so the dentist doesn’t need to buy such a device separately, but there is a need for reinforcing using (HVE) by increasing knowledge. Protective Mouth Rinse (PMR) with (0.2 %) chlorhexidine is also another method to prevent the spreading of splatter during dental care procedures. A study revealed that (PMR) can reduce the number of pathogens in the dental patient’s mouth if they used gargling agents [31]. (47.20 %) of participants in the current study were using this method (PMR) which was a low percentage. In another study, two-thirds of dental practitioners would ask their dental patients to use a (PMR) before starting the treatment [32]. This low percentage in the current study may be attributed to the expensive price of such rinse, low knowledge about the use, and the importance of this method. Aerosol control in general between participants was (55.1 %), this result is very important to be taken into consideration by the competent committees, because, most infectious diseases are transmitted by contaminated air inhalation especially nowadays while we are living the ghost of COVID-19. Significant differences in aerosol control among participants were attributed to the governorate variable (P-value<0.05). The T-test showed that dental clinics in Nablus district with means=0.51 are lower in applying aerosol control than those of Tulkam with a mean=0.61. On the other hand, there were significant differences in aerosol control among participants attributed to the years of experience variable ″P < 0.05″. The Post-Hoc test showed that the group of (≤5 years) with a mean=0.61 were the higher in applying aerosol control among others. Finally, in terms of this domain, there were significant differences in aerosol control among participants attributed to the ownership variable ″P < 0.05″. The Post-Hoc test showed that private clinics with mean=0.56 were the highest to apply aerosol control. This may indicate the desire of dental customers to receive their treatment in private clinics, which sometimes contributes to their income. But in the public and UNRWA clinics, any reviewers do not affect their income because there is no payment for treating and the income is constant. So, there is a need to find a policy and incentives to encourage public and UNRWA employees in order to improve the quality of the service.

### Accident management

The first aspect of accident management is having a protocol for dealing with sharp instruments during dental care procedures. According to CDC, accident management protocol, includes stopping the procedure, washing the affected area immediately with soap or disinfectant and water, using sterile water in case of exposure of mucous membrane, assessing the depth of injury, then checking the instrument, whether it was contaminated with blood or any body fluids, assessing the risk factors for the patient and the immunity status of the dentist for HBV and finally, taking of prophylaxis in the case of exposure to HBV, HIV, HCV ( Hepatitis C Virus) [33].

Nearly (50 %) of participants have such protocol, to deal with accidents during dental care procedures, this percentage is low compared with another one in which (81.0 %) of dentists had a clear protocol for needle stick emergency treatment and other sharps accidents [19]. The low percentage can be due to low knowledge or low awareness among dentists. The second important aspect of accident management was included in this study is having a puncture-resistant container for sharp instruments in the clinic. This study shows that a high percentage of the participants (90.60 %) have a puncture-resistant container for sharp instruments in their clinics. This result is better compared with another one in which (88.4 %) of dentists have puncture-resistant containers for sharp instruments [19]. The practitioners in this field reflect good knowledge, high awareness toward themselves and other people. Medical waste disposal is the third important aspect of accident management included in the current study. Mercury, silver, lead, blood, sharps, and chemicals should be managed as hazardous waste to protect the environment from environmental disasters. A very low percentage of participants in our study (24.90 %) has methods to dispose of their medical waste, this indicates a very big problem regarding cross-infection transmission for dentists and the community. The reasons for this problem should be dealt with seriously by the competent committees. In another study (81 %) of responses were non-regulated general medical waste procedure which produced within their dental offices, and the medical waste is disposed of in the general clinic trash [34]. The causes of such a problem may be because of low knowledge about the importance of methods to dispose of its medical wastes, no supervision, or the high cost of these methods. Our study also, revealed that (55.30 %) of the participants complied with accident management, this low percentage put dental practitioners in danger. In the same vein, (84.20 %) of participants ask their patients about their medical history. This result is better than the documented one in an Indian study in which (52 %) of Indian dentists had the habit of taking a medical history for all dental patients [35]. Although patients may not tell the dentist about the real infectious situation, the dentist should ask about the medical history of each patient in many ways to protect himself, his staff, and his clients. According to CDC guidelines, each accident in the clinic should be documented to prevent all sharp-related injuries and the transmission of blood-borne infections [36]. In this way, the dentist can also protect himself. Only (21.30 %) of participants document accidents which reflects a lack of knowledge and skills. Another study revealed that only (33.9 %) of HCWs documented their injuries [37].

As in all domains of this study, there were significant differences in accident management among participants attributed to the Nablus and Tulkarm variable in favor of the Tulkarm district ″P < 0.05″. The T-test showed that Tulkarm dental clinics with means=0.76 were more compliant to applying accident management in their clinics than Nablus dental clinics with a mean=0.65. Significant differences also were presented in accident management among participants attributed to the ownership variable ″P < 0.05″. The Post-Hoc test showed that private clinics with means=0.70 are applyed accident management more than other clinics (public and UNRWA). This highlighted that a private clinic dentist is very careful and keen not to have an accident in his/her clinic. Thus, that will keep both the clinic’s reputation and the clients. While in public and UNRWA clinics, the dentist gets a fixed salary, no strict health system that sues the dentist in case of accidents resulted from negligence, and irresponsibility toward patients and society due to the lack of knowledge and awareness.

### Limitations of the study

This questionnaire was distributed to dental practitioners during the peak period of COVID-19 pandemic in Palestine, in which all health care institutions were closed except urgent medical procedures. Thus, the author was unable to verify the authenticity of the answers, due to the inability to conduct an inspection and check-up.

## Conclusions

In conclusion, this study, which was done among dental clinics in Tulkarm and Nablus districts revealed a critical need for strict adherence and compliance to infection control protocol among dentists in both districts to prevent the transmission of infectious diseases in any health care setting.

### Recommendations

Evaluation of an actual infection control protocol of sampling tools, instruments, surfaces and culture them as well as checking their disinfection and sterilization status and evaluation of the patient’s confidence and perception of dental clinics regard infection control practices. In addition to the establishment of the infection control committee to plan, monitor and control, and also evaluate the infection control measures in oral health care settings. This suggested committee will also be responsible for improving, developing, and updating infection control strategies and standards, identifying training course’s needs and establishing training modules taking into consideration COVID 19 pandemic and the documentation of the medical history of patients.

## Material and method

### Study design

A descriptive, quantitative, and cross-sectional design was adopted in order to assess the level of knowledge and compliance about infection control measures in dental clinics.

### Study settings

This study has taken place in the dental clinics in Nablus and Tulkarm Governorates due to the proximity in the distance.

### Study population

A universal sample of dentists who were registered in the Palestinian dental association to practice dentistry in Nablus and Tulkarm districts have been recruited. The sample size was estimated at a 95 % Confidence Interval (C.I) accepting a 5% error margin using the sample size calculator (www.raosoft.com) for a population of nearly 690 dentists practicing in both districts [38]. The total number of the study population was 675 dentists. The study targeted a sample of 245 dentists, and therefore the ratio of the study sample to the total study population was 245/675 = 0.362 dentists. The dentists who were working in public dental clinics, and UNRWA (United Nations Relief and Works Agency For Palestine Refugees in The Near East) have been excluded to be taken obligatory in both districts (15 dentists) due to their low numbers. Based on the foregoing, the sample has been taken from each governorate according to the following formula: the number of dentists in the governorate×0.362 regardless of age, gender, educational level, and years of practice. The objectives of the indicators had been accomplished to be representative and generalized.

### Period of the study

The questionnaire was distributed between July and August 2020 which was the peak of COVID-19 (Coronavirus disease-2019) in Palestine via email to 265 dentists in Nablus and Tulkarm districts. Participants who did not respond to the first mailing were reminded 3 weeks later through an emailed memo.

### Study tool and variables

**Fig. 1:**
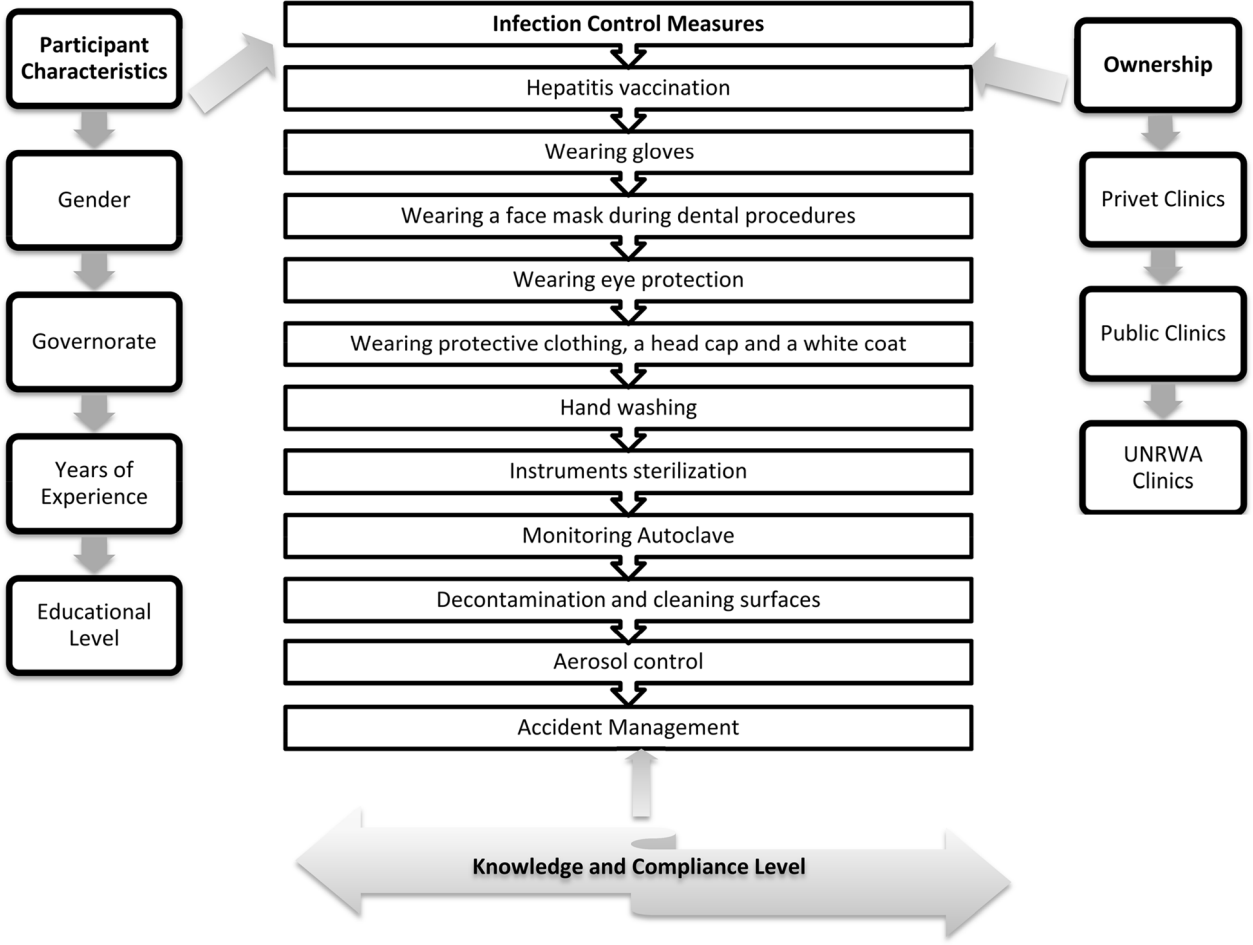
Conceptual Framework Model of the Study

 This study covered 10 issues of the basic guidelines for infection control in dental clinics, according to CDC (Center Of Disease Control And Prevention) and Palestinian infection and training protocols to assess the dentists’ knowledge and compliance with the basic guidelines of infection control in dental clinics. The questionnaire was comprising 63 questions. The first part of the questionnaire asks about the demographic profile. Then each participant has answered a series of questions about the infection control measures that they have been done regularly during clinical practice. The main variables that have been analyzed during this study were the infection control measurements using in dental clinics, knowledge, and compliances about/with these measures, see Fig. 1.

### Validity and reliability

To test the suitability of the current study method, a pilot study was carried out on a random sample of 20 dentists. The validity of the study tool was evaluated and modified by a group of experts and the coefficient of reliability (Cronbach’s alpha) was acceptable 0.753.

### Statistical analysis

Collected data was reviewed for completeness and accuracy. Initially, MS excel has been utilized for coding the data obtained through the questionnaire and resulting answers has been recorded and processed using the Statistic Package for the Social Sciences (IBM SPSS for Windows, Version 20.0). Descriptive statistics and bivariate analysis have been carried out using the Chi-square test to discuss the differences in infection control measures, knowledge, and compliance according to demographic characteristics. The question that has more than 80 % positive answers was judged as the highest score, moderate if positive answers were between 60 and 79 %; whereas, low if <60 % positive answers. One-way ANOVA, Post-Hock tests, and others have been used to compare means overall compliance by ownership of the clinic. Statistical significance has been set at ″p < 0.05″.

## Data Availability

The datasets of the current study are available from the corresponding author on reasonable request.

## Abbreviations

CDC: Center Of Disease Control And Prevention
COVID-19: Coronavirus Disease 2019
HBV: Hepatitis B Virus
HCV: Hepatitis C Virus
HIV: Human Immunodeficiency Virus
PPE: Patient Protective Equipment’s
SARS-CoV-2: Severe Acute Respiratory Syndrome Coronavirus
TB: Tuberculosis
UNRWA: United Nations Relief and Works Agency For Palestine Refugees in The Near East
WHO: World Health Organization
